# *In vitro* evaluation of the effect of different disinfectants on the biofilm of *Staphylococcus epidermidis* and *Staphylococcus aureus* formed on acrylic ocular prostheses

**DOI:** 10.1371/journal.pone.0240116

**Published:** 2020-10-12

**Authors:** Amália Moreno, Daniela Micheline dos Santos, Clóvis Lamartine de Moraes Melo Neto, André Luiz de Melo Moreno, André Pinheiro de Magalhães Bertoz, Marcelo Coelho Goiato

**Affiliations:** 1 Department of Oral Surgery, Pathology and Clinical Dentistry, Federal University of Minas Gerais (UFMG), School of Dentistry, Belo Horizonte, Minas Gerais, Brazil; 2 Department of Dental Materials and Prosthodontics, São Paulo State University (UNESP), School of Dentistry, Araçatuba, São Paulo, Brazil; 3 Oral Oncology Center, São Paulo State University (UNESP), School of Dentistry, Araçatuba, São Paulo, Brazil; 4 Brazilian Institute of Northern Education (IBEN), Manaus, Amazonas, Brazil; 5 Department of Pediatric and Social Dentistry, São Paulo State University (UNESP), School of Dentistry, Araçatuba, São Paulo, Brazil; The University of Jordan School of Pharmacy, JORDAN

## Abstract

The aim of this study was to evaluate the effect of disinfectants on the biofilm of *Staphylococcus aureus* and *Staphylococcus epidermidis* formed on the acrylic surface of ocular prostheses. In this study, 396 acrylic specimens were manufactured (50% for *Staphylococcus epidermidis*, and 50% for *Staphylococcus aureus*). For each bacterium, 66 specimens were subjected to biofilm formation on their surfaces for 24 hours, 66 specimens were subjected to biofilm formation on their surfaces for 48 hours, and 66 specimens were subjected to biofilm formation on their surfaces for 72 hours. Then, they were divided into groups according to disinfection method (n = 6): sterile distilled water for 10, 15, 30 min, and 6 hours (control); soap for 30 min (NES30); Opti-Free for 30 min (OPF30) and 6 h (OPF6); Efferdent for 15 min (EFF15); and 0.5%, 2%, and 4% chlorhexidine for 10 min (0.5% CHX10, 2% CHX10, and 4% CHX10). After the treatments, the specimens were vortexed to release the biofilm and the counting of bacterial colonies was performed (CFU/mL). Three-way ANOVA and the Tukey-Kramer HSD test were used (α = 0.05). For *Staphylococcus epidermidis*, there was no significant difference between NES30, OPF30, and OPF6 with their respective control groups; nor between NES30, OPF30, and OPF6 themselves, regardless of the biofilm development period (*P* >0.05). For *Staphylococcus aureus*, there was no significant difference between NES30 and OPF30 with their control group; nor between NES30 and OPF30 themselves, regardless of the biofilm development period (*P* >0.05). For *Staphylococcus aureus*, OPF6 showed a significant reduction in the number of CFU/mL when compared with its control group, NES30, and OPF30, regardless of the biofilm development period (*P* <0.05). For both bacteria, 0.5% CHX10, 2% CHX10,4% CHX10, and EFF15 showed a significant reduction in the number of CFU/mL when compared with their control groups, NES30, OPF30, and OPF6, regardless of the biofilm development period (*P* <0.05). Therefore, EFF15 and CHX (0.5%, 2% and 4%) were effective in reducing *Staphylococcus epidermidis* and *Staphylococcus aureus* on acrylic surfaces. NES30 and OPF (30 and 6) are not recommended.

## Introduction

The ocular prosthesis is a modality of the maxillofacial prosthesis, and is considered an important treatment for patients who underwent total or partial loss of an eyeball due to trauma, cancer, or genetic defects [1]. An eye prosthesis has the function of restoring aesthetics, preventing eyelid deformation (preserving the palpebral muscle tone), protecting the anophthalmic cavity from smoke pollutants and dust, guiding the tear flow and preventing the accumulation of tear fluid in this cavity [1–3]. This type of prosthesis is also responsible for helping to improve the psychological factor and quality of life of the patient [1].

Acrylic resin (polymethyl methacrylate or PMMA) is the material of choice for ocular rehabilitation due to its ease of handling, good adaptation, satisfactory esthetics, and low cost [4]. When the acrylic ocular prosthesis is in position inside the anophthalmic cavity, some degree of "dead space" can be observed between the back surface of the prosthesis and the anophthalmic cavity tissue, especially when the ocular prosthesis is poorly adapted in this cavity [1, 5, 6]. According to Toribio et al. 2019, the lack of an ocular globe and the use of an ocular prosthesis produce several modifications in the biomechanics in the anophthalmic region. First, the bulbar conjunctiva is no longer swept by the eyelids [7]. Second, the artificial eye is a relatively large foreign body that can produce frictional irritation of the conjunctiva when the prosthesis moves [7]. Third, the "dead space" allows the accumulation of conjunctival debris, even in custom fitted prostheses [7]. In addition, the removal and placement of an ocular prosthesis in the anophthalmic cavity, and a poor prosthesis adjustment, can cause tissue irritation of the anophthalmic cavity [8]. These circumstances promote an increase of mucus in the socket, which in turn favors the growth of pathogenic microorganisms [7]. All these factors, combined with the presence of biofilm on the ocular prosthesis (due to poor hygiene), facilitate the development of bacterial infections [1, 5]. Thus, the disinfection of this type of prosthesis is essential to help prevent infections. According to Paranhos et al. 2007, disinfection of the ocular prosthesis is essential as it leads to a reduction in the number of microorganisms in the anophthalmic cavity, improving the comfort of ocular prosthesis users, and consequently their life quality [3].

One of the most important genera of pathogens in prosthetic infections is *Staphylococcus* [1, 9]. *Staphylococcus epidermidis* can adhere and proliferate on the surface of lenses and ocular prostheses made of PMMA [1, 9], secreting viscous extracellular matrix that protects it against antibiotics and host defense mechanisms [1]. *Staphylococcus aureus* lives principally on mucous surfaces and is considered one of the most versatile and dangerous human pathogens [1, 9]. These are two of the most prevalent species present in the anophthalmic cavity of ocular prosthesis users [1, 3, 5]. In addition, *Staphylococcus epidermidis* and/or *Staphylococcus aureus* can generate an infection of the anophthalmic cavity, thus edema, mucopurulent discharge, and hyperemia can be signals of this infection [5].

Some products have been studied to clean/disinfect the ocular prosthesis, such as neutral soap, chlorhexidine gluconate, alkaline peroxide, and multipurpose solution [3, 10, 11]. However, there are no studies that investigate the reduction of biofilm of *Staphylococcus aureus* and *Staphylococcus epidermidis* on the acrylic surface of ocular prostheses based on these disinfection methods. The aim of this study was to evaluate the effect of different disinfectants on the biofilm of *Staphylococcus aureus* and *Staphylococcus epidermidis* formed on the acrylic surface of ocular prostheses.

## Material and methods

### Preparation of acrylic resin specimens

**Fig 1:**
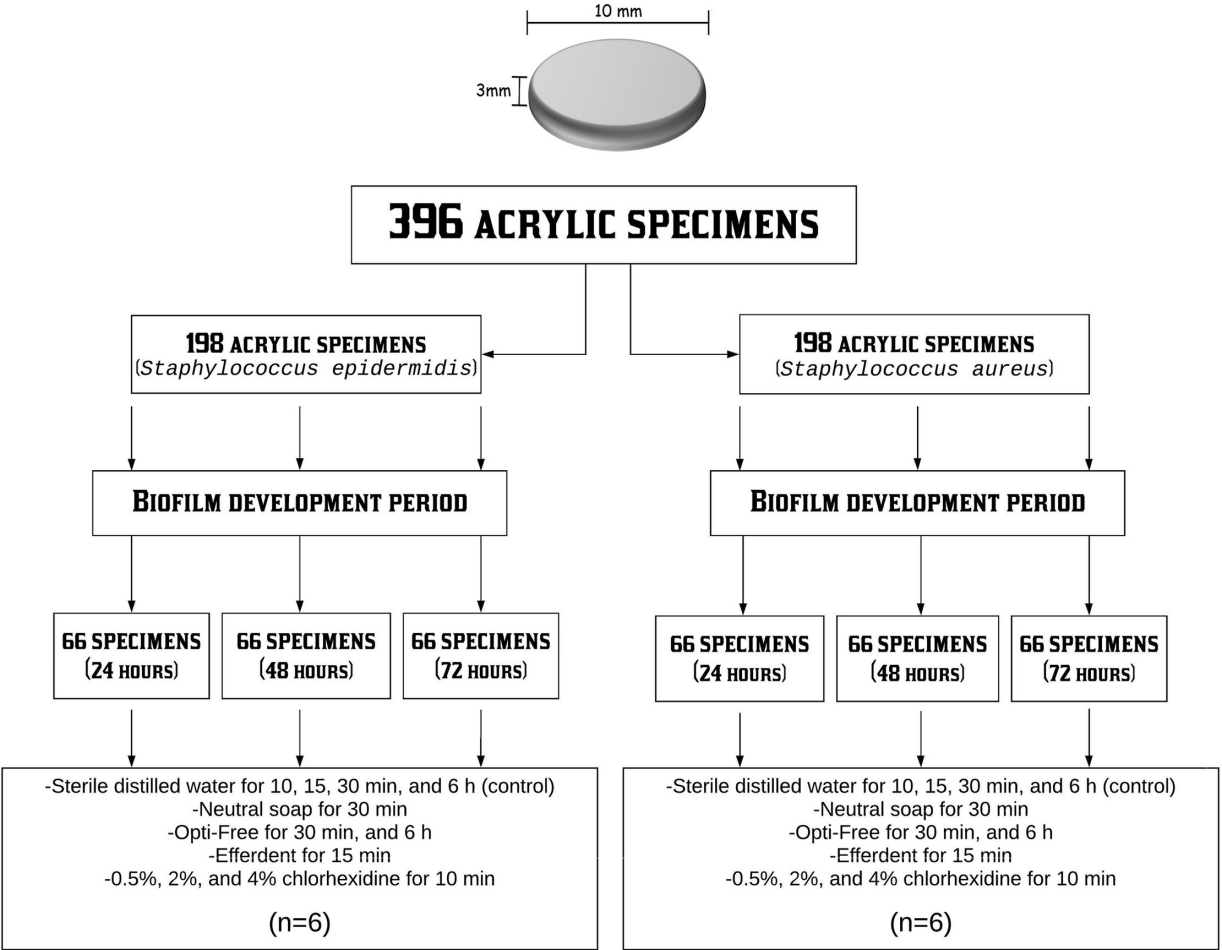
Distribution of groups.

In this study, 396 circular acrylic specimens with a diameter of 10 mm and a thickness of 3 mm were manufactured [4, 9]. Half of these specimens (198 specimens) were used for the formation of the *Staphylococcus epidermidis* biofilm, and the other half (198 specimens) for the formation of *Staphylococcus aureus* biofilm. For each bacterium, 66 specimens were subjected to biofilm formation on their surfaces for 24 hours, 66 specimens were subjected to biofilm formation on their surfaces for 48 hours, and 66 specimens were subjected to biofilm formation on their surfaces for 72 hours. Then, the specimens were randomly distributed to create the groups based on disinfection methods. (n = 6) **(Fig 1 and Table 1)**.

**Table 1:** Disinfectants used in this study.

Product	Color	Manufacturer	Chemical Composition
Neutral soap	Colorless	Johnson & Johnson, Brazil	Glycerin, polyethylene glycol sorbitan laurate, sodium tridecyl ether sulfate, cocamidopropyl betaine, Cocoanfocarboxiglicinato, Cetyl Alcohol Ethoxylated and propoxylated, lauryl polyglucose, ethoxylated lanolin, sodium lauryl ether carboxylate, polyethylene glycol distearate, fragrance, methylisothiazolinone and ethylchloroisothiazolinone, tetrasodium EDTA Acid citrus, Red Dye Food 1 and Water. Biodegradable formula.
Opti-Free Express multi-purpose solution	Colorless	Alcon, USA	Multi-Purpose Disinfecting Solution is a sterile, buffered, isotonic, aqueous solution containing sodium citrate, sodium chloride, boric acid, sorbitol, aminomethylpropanol, TETRONIC® 1304†, with edetate disodium 0.05%, POLYQUAD® (polyquaternium-1) 0.001% and ALDOX® (myristamidopropyl dimethylamine) 0.0005% preservatives.
Efferdent Original Denture Cleanser	Blue	Pfizer Consumer Healthcare, USA	According to Ingram et al. 2008, the ingredients of Efferdent (Pfizer) in alphabetical order are: FD&C Blue No.2, Ethylenediaminetetraacetic acid (EDTA), FD&C Green No.3, flavor, polytetrafloroethylene (Teflon, DuPont), potassium monopersulfate, sodium bicarbonate, sodium lauryl sulfoacetate, sodium perborate monohydrate, sodium saccharine, sodium sulfate, and sodium tripolyphosphate anhydrous [12]. The product has a pH of 9.5, weighs 2.1 g/tablet, and has a titratable O_2_ of 129–162 mg/tablet [12].
			The primary “active ingredient” in Efferdent is sodium perborate monohydrate (BO_3_Na•H_2_O). When BO_3_Na•H_2_O is added to water, it dissociates to form approximately 36% hydrogen peroxide and 64% sodium borate by weight [12]. A 2.1-g tablet of Efferdent dissolved in 250 mL of water should yield a solution of 0.174% hydrogen peroxide [12].
Chlorhexidine	Colorless	Sigma–Aldrich, USA	1,1'-hexamethylene bis [5-(p-chlorophenyl) biguanide] di-D-gluconate) in a base containing water, alcohol, glycerin, PEG-40 sorbitan diisostearate, flavor, sodium saccharin, and FD&C Blue No.1. Chlorhexidine gluconate product is a near neutral solution (pH range 5–7).

The standardization of the specimen dimensions was obtained with a metallic matrix containing 10 orifices with the dimensions of the future specimens [9]. This metallic matrix was fixed on a rectangular glass plate [9]. Then, the glass plate and the metallic matrix were placed in plaster Type IV (Durone, Dentsply, Brazil) in a microwave muffle (Nova OGP, Brazil) [9]. After the plaster crystallized, another glass plate was positioned over and affixed to the matrix. The other part of the muffle was positioned, screwed, and special plaster type IV (Durone, Dentsply) was poured over the last glass plate [9]. After the plaster crystallized, the muffle was opened for the insertion of the acrylic resin.

The No.1 heat-activated acrylic resin (white color) powder (Clássicos, Brazil) and the microwave-specific liquid (Onda-Cryl, Clássicos, Brazil) were mixed according to the manufacturer's recommendations. The acrylic resin was then added in the orifices of the matrix when it was in its plastic phase. After the addition of the resin, the muffle was pressed under a load of 1200Kgf (Hydraulic press—Maxx, VH, Brazil) for 2 minutes [9–11, 13, 14]. Subsequently, a 30 min bench polymerization was performed [9]. Afterwards, the resin was polymerized by microwave energy (BMS35, Brastemp, Brazil) with 1200W of potency for 10 minutes (initially, 3 minutes with 30% power, then 4 minutes without power (0%), and finally, 3 minutes with 60% power) [11, 13, 14]. After the acrylic resin polymerization, the muffle was opened and the specimens were removed.

The specimens were polished in an automated polishing machine (Ecomet 300 PRO Buehler, USA) with 120-grit sandpaper (Norton Abrasivos, Brazil) [15] under constant water irrigation at 300 rpm (revolutions per minute) [4]. Sandpaper was used on each side of each specimen until the Ra (roughness average) of the specimen was between 1 and 2μm [15]. Surface roughness was measured using a roughness meter (Dektak D-150, Veeco, USA) [4, 9]. The Ra values were measured using a cut‑off of 500 μm in a 12-second time constant [9]. Three readings were performed on each surface and the mean was calculated. The original values were given in Angstrom (Å) and were then transformed to μm [9]. Subsequently, each specimen was measured using a precision digital caliper (Mitutoyo, Japan) to ensure the proposed dimensions [10, 11].

The specimens were stored in deionized water inside a digital bacteriological incubator (CE-150/280I, CIENLAB Equipamentos Científicos Ltd, Brazil) at 37 ± 2°C for 50 ± 2 h, so as to hydrate them while residual monomers were eliminated [10, 11, 13, 14]. Then, the specimens were cleaned ultrasonically (Arotec, Odontobrás, Brazil) for 20 min with sterile deionized water to remove debris from the resin surface, dried and sterilized with ethylene oxide [9, 15].

### Inoculum and growth conditions

Strains of *Staphylococcus aureus* (ATCC 6538—slime-positive) and *Staphylococcus epidermidis* (ATCC 35984—slime-positive) were used. For the preparation of the inoculum, the microorganisms were kept at -70°C in solution containing 25% glycerol, and seeded in plates containing TSB culture medium (Tryptic Soy Broth Agar, Becton Dickinson and Company, USA) at 37°C [9, 16]. The culture was initiated in 5 mL of Brain Heart Infusion Broth (BHI) (Becton Dickinson and Company, USA) and kept growing at 37°C for 18 hours to obtain cells in the exponential phase [16]. After this period, the optical density was measured by spectrophotometer in order to establish the concentration of 0.01 at OD600. The amount of inoculated culture was calculated to obtain approximately 1 × 10^8^ CFU/mL (colony forming units per mL).

### Biofilm development

The acrylic resin specimens were removed from the sterilization envelope with the aid of a sterile forceps. Each acrylic specimen was individually placed in an orifice of 6-well plates, so that inside each orifice there was 6 mL of Tryptic Soy without dextrose (Becton Dickinson and Company, USA), supplemented with 1% glucose. Then, the inoculum of *Staphylococcus epidermidis* or *Staphylococcus aureus*, with approximately 10^8^ CFU/mL, was added to the medium. The organisms were grown at 37°C while agitated (100 rpm) in an orbital shaker (model C24, Incubator Shaker, Edison, USA). Three phases of biofilm development were examined: 24 hours, 48 hours [9], and 72 hours. Every 24h, each acrylic resin specimen was removed with sterile forceps and washed gently with 6 mL of sterile phosphate buffered saline (PBS) twice, to remove loosely adhered bacteria. Then, all specimens were placed into 6-well plates (one specimen in each orifice) with fresh medium.

### Treatment of specimens by the disinfection solution

After each biofilm development period (24, 48, and 72 hours), the specimens were immediately disinfected. The disinfection treatment was carried out inside the flow chamber, removing the specimens from the 6-well plates and immersing each one (individually) in 5 mL of its respective disinfectant substance (according to the group of each specimen). The disinfectants used were based on the studies by Moreno et al. 2012 and Moreno et al. 2013 [10, 11]. The groups (n = 6) were formed according to the disinfection method **(Fig 1)**:

- sterile distilled water (control) for 10 min, 15 min, 30 min, and 6 hours (CTL10, CTL15, CTL30, and CTL6);
- neutral soap for 30 min (NES30);
- Opti-Free Express multipurpose solution for 30 min (OPF30) and 6 h (OPF6) [the 6-hour period was based on the manufacturer's recommendation];
- 0.5%, 2% and 4% chlorhexidine gluconate for 10 min (0.5% CHX10, 2% CHX10, and 4% CHX10) [13, 14]. The 0.5%, 2%, and 4% CHX10 solutions were prepared on the same day of the experiment;
- initially, the tablet of Efferdent Original Denture Cleanser was added into ~250mL of sterile water [12]. Posteriorly, the specimen was immersed in 5mL of water with Efferdent Original Denture Cleanser for 15 min (EFF15) [13, 14].

Subsequently, each specimen was washed with PBS for 15 seconds.

### Counting bacterial colonies immediately after disinfection

After the cleansing procedure and washing in sterilized PBS, each acrylic resin specimen was immersed in 1 mL of PBS and vortexed (3,200 rpm) (Fisher Scientific, USA) to break down the structure of the biofilm formed on its surface. The vortex procedure was performed three times, for 1 minute each time, in a 4°C environment (the tubes rested on ice for 2 minutes between each stirring) to release the biofilm adhered to the specimens. Then the specimens were removed from the tubes, leaving only the solutions, which were diluted and plated in triplicate on BHI agar. The plates were incubated at 37°C under aerobic conditions for 24h. Bacterial colonies were counted using a colony counter (Fisher Scientific, USA) and then the number of CFU/mL was determined.

### Statistical analyses

All data were analyzed using the Statistical Package for Social Sciences 21.0 (IBM Corp., USA). The 3-way repeated-measures analysis of variance (ANOVA) was performed to determine if there were significant differences between the strain, disinfectants, and biofilm development period. Subsequently, the Tukey-Kramer HSD test was used. The significance level was 5%.

## Results

Based only on the ´´strain” factor, the number of CFU/mL of *Staphylococcus epidermidis* was statistically significantly higher when compared with the number of CFU/mL of *Staphylococcus aureus* (*P* <0.0001) **(Table 2)**. In addition, based only on the ´´biofilm development period” factor, there was a statistically significant increase in the number of CFU/mL over time (*P* <0.0001) **(Table 2).**

**Table 2:** Results of 3-way repeated-measures ANOVA.

Factors	Degree of freedom	Sum of squares	Mean square	F value	P value
Strain	1	1.28E+21	1.28E+21	316.617	< 0.0001*
Disinfectant	10	1.17E+21	1.17E+20	29.078	< 0.0001*
Biofilm development period	2	2.50E+21	1.25E+21	309.474	< 0.0001*
Strain x Disinfectant	10	8.37E+20	8.37E+19	20.745	< 0.0001*
Strain x Biofilm development period	2	1.82E+21	9.08E+20	224.940	< 0.0001*
Strain x Disinfectant x Biofilm development period	20	1.66E+21	8.28E+19	20.514	< 0.0001*
Error	330	1.33E+21	4.04E+18		
Total	395	1.18E+22			

* denotes a statistically significant difference (*P* <0.05).

### Staphylococcus epidermidis

**Fig 2:**
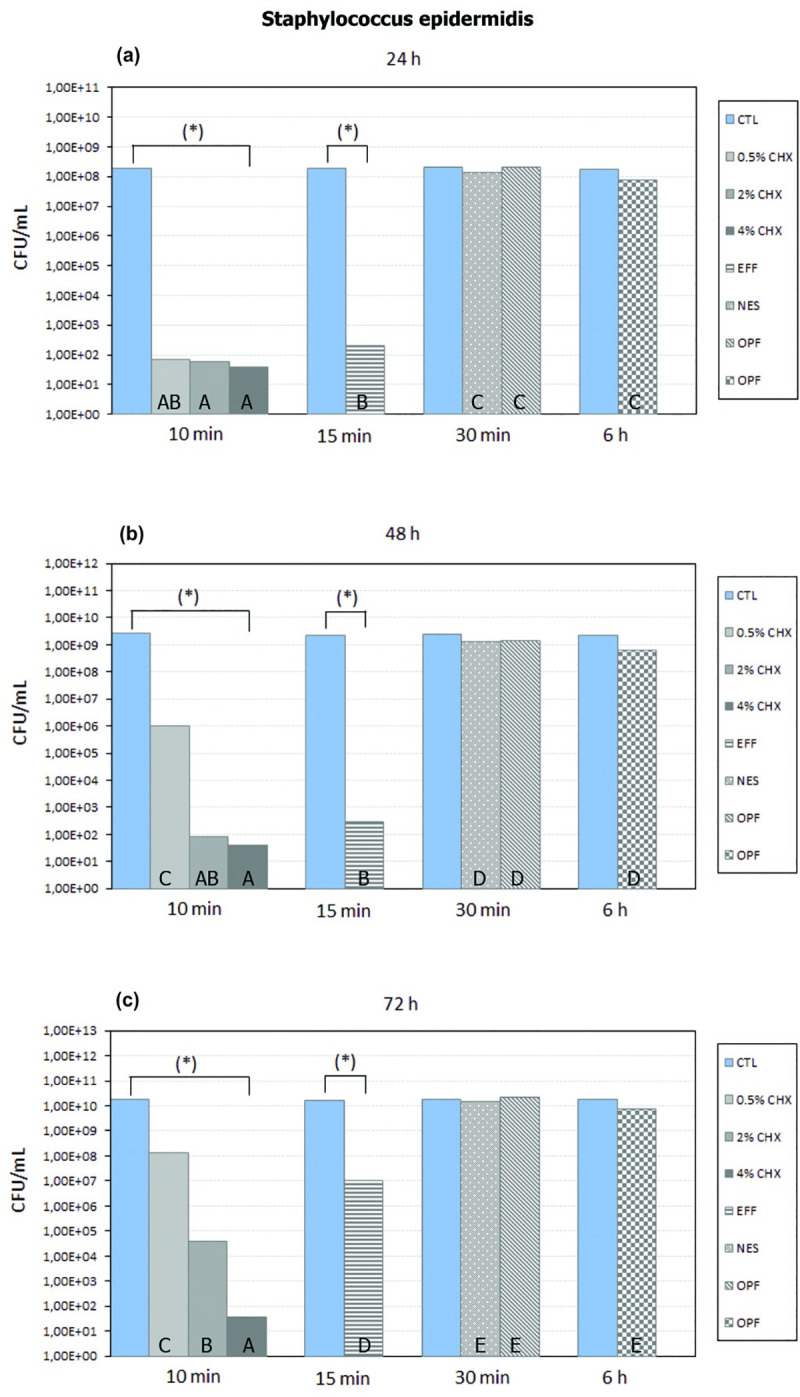
Tukey-Kramer HSD test with a level of significance of 5%. Mean (number of CFU/mL of *Staphylococcus epidermidis*) of each control group and each disinfectant group in 24 h (a), 48 h (b) and 72 h (c) of biofilm development. For a comparison only between control group and disinfectant group in each biofilm development period (individually), * means that there is a statistical difference between control group and disinfectant group (*P* <0.05). For a comparison only between disinfectant groups in each biofilm development period (individually), different letters represent a statistically significant difference (*P* <0.05).

The 0.5% CHX10, 2% CHX10, 4% CHX10, and EFF15 groups showed a significant reduction in the number of CFU/mL of *Staphylococcus epidermidis* compared with their respective control groups, regardless of the biofilm development period (*P* <0.05) **(Fig 2).**

The NES30, OPF30, and OPF6 groups showed no statistically significant difference in the number of CFU/mL of *Staphylococcus epidermidis* when compared with their respective control groups, regardless of the biofilm development period (*P* >0.05) **(Fig 2)**.

There was no statistically significant difference in the number of CFU/mL of *Staphylococcus epidermidis* between the OPF30, OPF6, and NES30 groups (*P* >0.05), regardless of the biofilm development period **(Fig 2)**. The 0.5% CHX10, 2% CHX10, 4% CHX10, and EFF15 groups showed a greater statistically significant reduction in the number of CFU/mL of *Staphylococcus epidermidis* compared with NES30, OPF6, and OPF30, regardless of the biofilm development period (*P* <0.05) **(Fig 2)**.

After 24 hours of biofilm development: **I)** there was no statistically significant difference between the 0.5% CHX10, 2% CHX10, and 4%CHX10 groups (*P*>0.05); **II)** the 2% CHX10 and 4% CHX10 groups showed a significant reduction in the number of CFU/mL of *Staphylococcus epidermidis* compared with the EFF15 group (*P* <0.05); and **III)** there was no statistically significant difference between the 0.5% CHX10 and EFF15 groups (*P*>0.05) **(Fig 2)**.

After 48 hours of biofilm development: **I)** there was no statistically significant difference between the 2% CHX10 and 4% CHX10 groups (*P*>0.05); **II)** there was no statistically significant difference between the EFF15 and 2% CHX10 (*P*>0.05) groups; **III)** the 4% CHX10 group showed a significantly greater reduction in the number of CFU/mL of *Staphylococcus epidermidis* compared with the EFF15 and 0.5% CHX10 groups (*P* <0.05); and **IV)** the 2% CHX10, 4% CHX10, and EFF15 groups showed a significantly greater reduction in the number of CFU/mL of *Staphylococcus epidermidis* compared with the 0.5% CHX10 group (*P* <0.05) **(Fig 2)**.

After 72 hours of biofilm development: **I)** the 4% CHX10 group showed a significantly greater reduction in the number of CFU/mL of *Staphylococcus epidermidis* than the 0.5% CHX10, 2% CHX10, and EFF15 (*P* <0.05); **II)** the 2% CHX10 group showed a significantly greater reduction in the number of CFU/mL of *Staphylococcus epidermidis* than the 0.5% CHX10 and EFF15 (*P* <0.05); and **III)** the EFF15 group showed a significantly greater reduction in the number of CFU/mL of *Staphylococcus epidermidis* than the 0.5% CHX10 group (*P* <0.05) **(Fig 2)**.

Comparing the biofilm development periods based on the 0.5% CHX10 group, it is possible to verify that in the period of 24 hours, there was a significantly greater reduction in the number of CFU/mL of *Staphylococcus epidermidis* when compared to the periods of 48 and 72 hours (*P* <0.05). In addition, in the period of 48 hours, there was a significantly greater reduction in the number of CFU/mL of *Staphylococcus epidermidis* when compared to the period of 72 hours, based on the 0.5% CHX10 group (*P* <0.05).

Comparing the biofilm development periods based on the EFF15 group or 2% CHX10 group, it is possible to verify that in the periods of 24 and 48 hours, there was a significantly greater reduction in the number of CFU/mL of *Staphylococcus epidermidis* when compared to the period of 72 hours (*P* <0.05). In addition, based on the EFF15 group or 2% CHX10 group, there was no difference between the periods of 24 and 48 hours (*P* >0.05).

Comparing the biofilm development periods, based on the 4% CHX10 group, there was no significant statistical difference in the number of CFU/mL of *Staphylococcus epidermidis* between the periods of 24, 48, and 72 hours (*P* >0.05).

### Staphylococcus aureus

**Fig 3:**
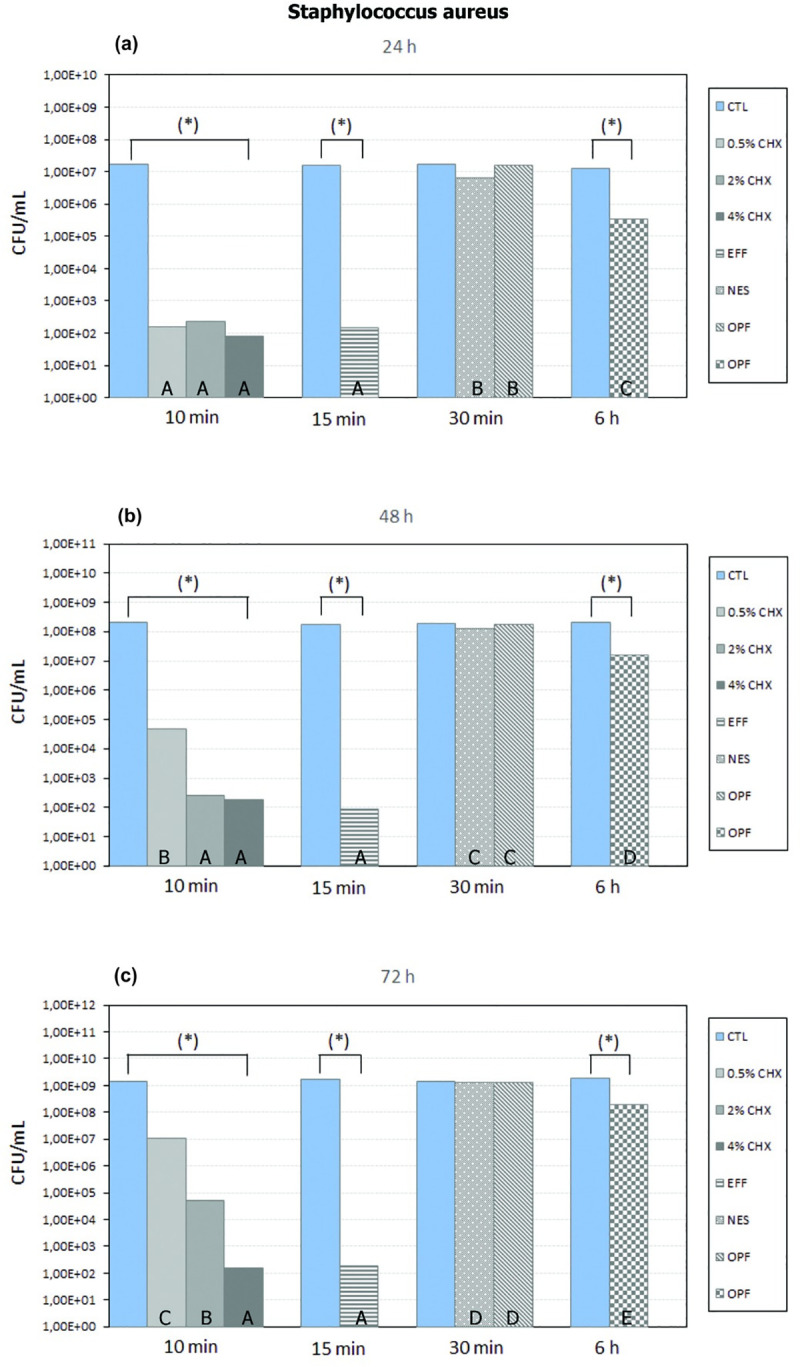
Tukey-Kramer HSD test with a level of significance of 5%. Mean (number of CFU/mL of *Staphylococcus aureus*) of each control group and each disinfectant group in 24 h (a), 48 h (b) and 72 h (c) of biofilm development. For a comparison only between control group and disinfectant group in each biofilm development period (individually), * means that there is a statistical difference between control group and disinfectant group (*P* <0.05). For a comparison only between disinfectant groups in each biofilm development period (individually), different letters represent a statistically significant difference (*P* <0.05).

The 0.5% CHX10, 2% CHX10, 4% CHX10, EFF15, and OPF6 groups showed a significant reduction in the number of CFU/mL of *Staphylococcus aureus* compared with their respective control groups, regardless of the biofilm development period (*P* <0.05) **(Fig 3).**

The NES30 and OPF30 groups showed no statistically significant difference in the number of CFU/mL of *Staphylococcus aureus* when compared with their control group, regardless of the biofilm development period (*P* >0.05) **(Fig 3)**.

There was no statistically significant difference in the number of CFU/mL of *Staphylococcus aureus* between the OPF30 and NES30 groups, regardless of the biofilm development period (*P* >0.05) **(Fig 3)**. In addition, OPF6 showed a greater statistically significant reduction of *Staphylococcus aureus* when compared with the OPF30 and NES30 groups, regardless of the biofilm development period (*P* <0.05) **(Fig 3)**. The 0.5% CHX10, 2% CHX10, 4% CHX10, and EFF15 groups showed a greater statistically significant reduction of *Staphylococcus aureus* compared with the NES30, OPF6, and OPF30 groups, regardless of the biofilm development period (*P* <0.05) **(Fig 3)**.

After 24 hours of biofilm development, there was no statistically significant difference between the 0.5% CHX10, 2% CHX10, 4% CHX10, and EFF15 groups (*P* >0.05) **(Fig 3)**.

After 48 hours of biofilm development: **I)** there was no statistically significant difference between the 2% CHX10, 4% CHX10, and EFF15 groups (*P* >0.05); and **II)** the 2% CHX10, 4% CHX10, and EFF15 groups showed a significantly greater reduction in the number of CFU/mL of *Staphylococcus aureus* compared with the 0.5% CHX10 group (*P* <0.05) **(Fig 3)**.

After 72 hours of biofilm development: **I)** there was no statistically significant difference between the 4% CHX10 and EFF15 groups (*P*>0.05); **II)** the 4% CHX10 and EFF15 groups showed a significantly greater reduction in the number of CFU/mL of *Staphylococcus aureus* compared with the 0.5% CHX10 and 2% CHX10 groups (*P* <0.05); and **III)** the 2% CHX10 group showed a significantly greater reduction in the number of CFU/mL of *Staphylococcus aureus* compared with the 0.5% CHX10 group (*P* <0.05) **(Fig 3)**.

Comparing the biofilm development periods based on the 0.5% CHX10 group, it is possible to verify that in the period of 24 hours, there was a significantly greater reduction in the number of CFU/mL of *Staphylococcus aureus* when compared to the periods of 48 and 72 hours (*P* <0.05). In addition, in the period of 48 hours, there was a significantly greater reduction in the number of CFU/mL of *Staphylococcus aureus* when compared to the period of 72 hours, based on the 0.5% CHX10 group (*P* <0.05).

Comparing the biofilm development periods based on the 2% CHX10 group, it is possible to verify that in the periods of 24 and 48 hours, there was a significantly greater reduction in the number of CFU/mL of *Staphylococcus aureus* when compared to the period of 72 hours (*P* <0.05). In addition, based on the 2% CHX10 group, there was no difference in the number of CFU/mL of *Staphylococcus aureus* between the periods of 24 and 48 hours (*P* >0.05).

Comparing the biofilm development periods based on the 4% CHX10 group or EFF15 group, there was no statistically significant difference in the number of CFU/mL of *Staphylococcus aureus* between the periods of 24, 48, and 72 hours (*P* >0.05).

## Discussion

This study simulated in vitro development of biofilm that can be found on the surface of an acrylic ocular prosthesis that is in contact with the anophthalmic cavity tissue.

An Ra ≤ 0.2 μm is clinically acceptable for the roughness of an acrylic resin prosthesis [17] because it hinders microbial adhesion [18]. However, an Ra > 0.2 μm facilitates microbial adhesion [18]. Therefore, in this study, the surface of the acrylic resin specimens was favorable for the microbial adhesion, due to their surface roughness between 1 and 2μm.

The present study showed that the number of CFU/mL of *Staphylococcus epidermidis* was statistically significantly higher when compared with the number of CFU/mL of *Staphylococcus aureus* (*P* <0.0001), based only on the ´´strain” factor **(Table 2)**. This difference may have occurred due to the fact that *Staphylococcus aureus* prefers to adhere to a metallic surface in comparison with the surface of a polymer (e.g., PMMA) [19, 20]. In addition, *Staphylococcus epidermidis* prefers to adhere to PMMA compared with a metallic surface [20]. Therefore, the greater preference of *Staphylococcus epidermidis* for PMMA would explain the greater number of CFU/mL of this microorganism on the PMMA specimens, when compared with the *Staphylococcus aureus* [20].

In the literature, it is possible to verify that mechanical methods (e.g., brushing) generally are not indicated for the hygiene of acrylic ocular prostheses [3, 21–24], since the surface of the prosthesis can be scratched or roughened, facilitating the adhesion of microorganisms. Therefore, the method of disinfection must be efficient in reducing the number of microorganisms on an acrylic eye prosthesis. In various articles, non-abrasive soap is the method most commonly indicated to disinfect/clean the surface of acrylic ocular prostheses [3, 21–24]. In addition, multiuse solutions (e.g., OPF) can also be indicated [24]. However, in the present study, for the *Staphylococcus epidermidis*, the NES30, OPF30, and OPF6 methods did not demonstrate a significant statistical difference in the number of CFU/mL in comparison with their respective control groups, independent of the period of biofilm development **(Fig 2)** (*P* >0.05). For *Staphylococcus aureus*, the NES30 and OPF30 methods also did not demonstrate a significant statistical difference in the number of CFU/mL in comparison with their control group, independent of the period of biofilm development **(Fig 3)** (*P*>0.05). These situations contradict the use of these products (NES30, OPF30, and OPF6) as disinfection methods of acrylic ocular prostheses. In addition, despite OPF6 having demonstrated a significant reduction in the number of CFU/mL of *Staphylococcus aureus* compared with its control group (*P* <0.05), independent of the period of biofilm development **(Fig 3)**, this reduction occurred after 6 hours of disinfection, which makes OPF6 inviable for the patient due to the long time necessary for the disinfection. It is worth highlighting that OPF6 did not statistically significantly reduce the number of CFU/mL of *Staphylococcus epidermidis*, independent of the period of biofilm development (*P* >0.05), which reinforces its contraindication by the fact of not being efficient against both microorganisms.

The 0.5% CHX10, 2% CHX10, 4% CHX10, and EFF15 methods demonstrated a statistically significant reduction in the number of CFU/mL of *Staphylococcus epidermidis* and *Staphylococcus aureus* in comparison with their respective control groups, independent of the period of biofilm development **(Figs 2 and 3)** (*P* <0.05). In addition, the 0.5% CHX10, 2% CHX10, 4% CHX10, and EFF15 methods demonstrated a significantly greater reduction in the number of CFU/mL compared with the OPF30, NES30, and OPF6 methods, independent of the period of biofilm development for *Staphylococcus epidermidis* and *Staphylococcus aureus* **(Figs 2 and 3)** (*P* <0.05). According to Paranhos et al. 2013, the efficacy of a disinfectant depends mainly on its penetration into the biofilm [25]. Therefore, the NES30 and OPF (6 and 30) disinfectants presumably did not penetrate, or did not adequately penetrate, the Staphylococcus epidermidis or Staphylococcus aureus biofilm, in comparison with other methods evaluated.

Some authors can explain the action of EFF15 and 0.5%, 2% and 4% CHX10. According to Cruz et al. 2011, when the effervescent tablet (e.g., EFF) is dissolved in water, it becomes an alkaline hydrogen peroxide, which decomposes and releases small bubbles of oxygen with the mechanical action of detaching the biofilm from the surface of the acrylic prosthesis [26], in addition to having an antimicrobial action [27]. Also, the mechanical action of oxygen can remove debris and stains from the surface of the prosthesis [27]. According to James et al. 2017, the positively charged (cationic) CHX molecule binds to the negatively charged microbial cell wall and interferes with the osmotic equilibrium of the microorganism [28]. At low concentrations, CHX is bacteriostatic, which causes leakage of low molecular weight substances from the microbial cell and inhibits microbial reproduction [28]. At higher concentrations, CHX is bactericidal, which causes cell death by precipitating the cytoplasmic contents of the microbial cell [28].

Moreno et al. 2012 and Moreno et al. 2013 evaluated hardness, roughness, and color of acrylic resins used for ocular protheses after disinfection with different products [10, 11]. One of the resins used was a translucent that was painted with an oil paint to simulate an iris (Onda-Cryl, Clássicos, Brazil), and the other was a white resin which simulated the sclera (Onda-Cryl, Clássicos, Brazil) [10, 11]. Moreno et al. 2012 and Moreno et al. 2013 performed the same disinfection protocols, in a manner where the samples of the NES, OPF, and 4% CHX groups were disinfected daily, employing manual friction with gauze for 1 min, and the samples of the EFF group (Pfizer Consumer Healthcare) were immersed for 15 min in the EFF solution, 3 times a week [10, 11] The total time of these studies was 120 days [10, 11]. According to Moreno et al. 2013, NES, EFF, OPF, and 4% CHX can increase roughness and reduce the Knoop microhardness of an acrylic resin [11]. Despite this, all of the increases in roughness of the NES, EFF, OPF, and 4% CHX groups after 120 days were clinically acceptable (<0.2 μm) [11, 17]. Moreno et al. 2013 also verified that there was no significant statistical difference in the microhardness values between the NES and EFF groups [11]. In the same study, despite the significant reduction in microhardness in all groups after the disinfection protocols (NES, EFF, OPF, and 4% CHX) [11], all of the values were clinically acceptable (>15 Knoop units) at the end of the experiment [11, 13]. In relation to alteration of color, it was observed that after 120 days, the NES, OPF, EFF, and 4% CHX methods generated a significant increase in the color values (ΔE) of the acrylic irises [10], however, all of these color changes were inferior to 3.7 (clinically acceptable) [29]. In addition, there was no significant statistical difference for color change comparing the disinfectants [10]. Goiato et al. 2013 and Goiato et al. 2013 evaluated specimens of acrylic resin (Onda-Cryl, Clássicos) after a total of 2000 cycles in a thermo cycler (alternated 30s baths with a temperature between 5 ±1 and 55 ±1°C) in association with disinfection 3 times per week for 60 days [13, 14]. In these studies [13, 14], there were 2 groups, in which one group of specimens was immersed in EFF (Pfizer Consumer Healthcare) for 15 min, and the other group of specimens was immersed in 4% CHX for 10 min [13, 14]. It was observed, at the end of the experiment, that the color alterations (ΔE < 3.7) and Knoop microhardness (>15 Knoop units) of the specimens were clinically acceptable [13, 14, 29]. It is worth mentioning that 2000 cycles are equivalent to 2 years of use of a complete denture [14], in a manner which could also represent 2 years of use of an ocular prosthesis. Based on these results [10, 11, 13, 14] and the results of the present study, the EFF15, 4% CHX10, and potentially the 0.5% and 2% CHX10 methods, are clinically valid options for the disinfection of acrylic ocular prostheses.

In relation to 0.5% CHX10, 2% CHX10, 4% CHX10, and EFF15, based on the evaluation in each period (individually), and based on the comparison between periods of biofilm development, the 0.5% CHX10 would be more recommended for the daily disinfection of ocular prostheses (every 24 hours). This recommendation would be based on the fact that, after 24 hours of biofilm development, there was no difference between the 0.5% CHX10 group and the 4% CHX10, 2% CHX10 and EFF15 groups, for *Staphylococcus epidermidis* and *Staphylococcus aureus* **(Figs 2 and 3)** (*P* >0.05). In addition, after 48 and 72 hours of biofilm development, the 2% CHX10, 4% CHX10, and EFF15 groups demonstrated a significant greater reduction in the number of CFU/mL of *Staphylococcus epidermidis* and *Staphylococcus aureus* compared with the 0.5% CHX10 group **(Figs 2 and 3)** (P <0.05). Another important situation is that, in the comparison between periods for the 0.5% CHX10 group, it was possible to verify, for example, that after 48 hours of biofilm development, there was a significant increase in the number of CFU/mL of *Staphylococcus epidermidis* and *Staphylococcus aureus* compared with the period of 24 hours (P <0.05). For disinfection of ocular prostheses every 48 hours, the 2% CHX10 or EFF15 would be the most recommended method. This can be suggested since there was no significant difference in the number of CFU/mL of *Staphylococcus epidermidis* and *Staphylococcus aureus* comparing the periods of 24 with 48 hours of biofilm development, based on 2% CHX10 or EFF15 (*P* >0.05). In addition, despite the 4% CHX10 group having showed a significantly greater reduction when compared with the EFF15 group after 48 hours of biofilm development for *Staphylococcus epidermidis* (P <0.05), there was no difference between the 2% CHX10 and 4% CHX10 groups or between the 2% CHX10 and EFF15 groups **(Fig 2)** (*P* >0.05). It is important to note that for *Staphylococcus aureus* there was no difference between the 2% CHX10, EFF15, and 4% CHX10 groups after 48 hours of biofilm development **(Fig 3)** (*P* >0.05). For disinfection of ocular prostheses every 72 hours, the 4% CHX10 would be the most recommended. This can be suggested since in the comparison between the periods of biofilm development (24, 48 and 72 hours), there was no significant difference in the number of CFU/mL of *Staphylococcus epidermidis* and *Staphylococcus aureus*, based on the 4% CHX10 group (*P* >0.05). In addition, for both microorganisms, the 4% CHX10 group demonstrated the best result, being the greatest reduction of both microorganisms after 72 hours of biofilm development. It is worth highlighting that the 2% CHX10, EFF15 or 4% CHX10 could also be recommended every 24 hours. However, as the 0.5% CHX10 demonstrated results equivalent to 2% CHX10, 4% CHX10, and EFF15 after 24 hours of biofilm development for both microorganisms, it would not be necessary to indicate another more effective method (2% CHX10, 4% CHX10, or EFF15) for daily disinfection. The same applies to the 4% CHX10, which could be recommended every 48 hours. However, the 2% CHX10 or EFF15 was effective after 48 hours of biofilm development for both microorganisms. Therefore, possibly with these suggestions, alterations of color, hardness, and roughness of an ocular prosthesis would be smaller in the long term.

Based only on the ´´biofilm development period” factor **(Table 2)**, there was a statistically significant increase in the number of CFU/mL over time (*P* <0.0001). This may suggest that the longer the biofilm develops, the greater its resistance to a disinfectant could be. According to Paranhos et al. 2013, the degree of biofilm development is a barrier to disinfection because it can make the penetration of a disinfectant into the biofilm slow and partial, most of the time [25]. Therefore, after the manufacturing of an ocular prosthesis, it is primordial that the dentist explains the importance of its daily disinfection (e.g., using 0.5% CHX10), or at most every 3 days (using 4% CHX10), to the patient, based on this study. Song et al. 2006 conducted a satisfaction survey related to users of ocular prostheses [30]. In the survey [30], although 84.6% of the 78 evaluated patients responded that they cleaned their prosthesis with water daily, most patients did not know exactly how to disinfect it [30]. This could have occurred, for example, due to the lack of knowledge of the dentists as how to adequately inform their patients about disinfection methods of ocular prostheses. This situation demonstrates the importance of the present study for dentists.

Although the results of the EFF15, 0.5% CHX10, 2% CHX10, and 4% CHX10 methods having been excellent in the present study, more studies are necessary to evaluate these methods of disinfection in other microorganisms that can also be encountered in the anophthalmic cavity. It is also recommended that brands of other alkaline peroxide companies be tested. In addition, more studies must be performed testing the physical and mechanical properties of other brands of acrylic resin after the application of different protocols of disinfection, during periods of time longer than 60 and 120 days.

## Conclusion

EFF15 and CHX (0.5%, 2%, and 4%) were effective in reducing the number of CFU/mL of *Staphylococcus epidermidis* and *Staphylococcus aureus* on acrylic resin surfaces. NES30 and OPF (30 and 6) are not recommended for disinfecting ocular prostheses.
